# Photoluminescence of Two-Dimensional MoS_2_ Nanosheets Produced by Liquid Exfoliation

**DOI:** 10.3390/nano13131982

**Published:** 2023-06-30

**Authors:** Mikhail Y. Lukianov, Anna A. Rubekina, Julia V. Bondareva, Andrey V. Sybachin, George D. Diudbin, Konstantin I. Maslakov, Dmitry G. Kvashnin, Olga G. Klimova-Korsmik, Evgeny A. Shirshin, Stanislav A. Evlashin

**Affiliations:** 1 Skolkovo Institute of Science and Technology, 121205 Moscow, Russia; mikhail.lukianov@skoltech.ru (M.Y.L.);; 2 Department of Physics, Lomonosov Moscow State University, 119991 Moscow, Russia; 3 Department of Chemistry, Lomonosov Moscow State University, 119991 Moscow, Russia; 4 Institute of Nanotechnology of Microelectronics of the Russian Academy of Sciences, 119991 Moscow, Russia; 5 Emanuel Institute of Biochemical Physics of the Russian Academy of Sciences, 119334 Moscow, Russia; 6 World-Class Research Center “Advanced Digital Technologies”, State Marine Technical University, 190121 Saint Petersburg, Russia

**Keywords:** transition metal dichalcogenides (TMDs), MoS_2_, liquid-phase exfoliation (LPE), photoluminescence

## Abstract

Extraordinary properties of two-dimensional materials make them attractive for applications in different fields. One of the prospective niches is optical applications, where such types of materials demonstrate extremely sensitive performance and can be used for labeling. However, the optical properties of liquid-exfoliated 2D materials need to be analyzed. The purpose of this work is to study the absorption and luminescent properties of MoS_2_ exfoliated in the presence of sodium cholate, which is the most often used surfactant. Ultrasound bath and mixer-assisted exfoliation in water and dimethyl sulfoxide were used. The best quality of MoS_2_ nanosheets was achieved using shear-assisted liquid-phase exfoliation as a production method and sodium cholate (SC) as a surfactant. The photoluminescent properties of MoS_2_ nanosheets varied slightly when changing the surfactant concentrations in the range C(SC) = 0.5–2.5 mg/mL. This work is of high practical importance for further enhancement of MoS_2_ photoluminescent properties via chemical functionalization.

## 1. Introduction

Two-dimensional materials are considered an extremely promising solution for the future generation of electronics [1]. In particular, transition metal dichalcogenides (TMDs) have attracted considerable attention in the research community due to their unique mechanical [2,3], optical [4,5,6,7,8,9,10] and electrical properties [11,12]. The natural abundance of TMDs, as well as their tunable band gap in the visible and near-infrared ranges [13,14], make them ideal candidates for future optoelectronic devices. The MoS_2_ band gap changes from an indirect band gap of 1.1 eV in the bulk form to a direct band gap of 1.9 eV as the thickness decreases to a monolayer [15], which gives a great advantage over graphene [16]. Due to such bandgap transition, molybdenum disulfide is used as the main material for optical sensors, field-effect transistors [17,18], single-photon emitters [19,20,21], wearable electronic devices based on the piezophototronic effect [22] and ultrasensitive photodetectors [12], as well as for many other potential applications [23].

For the mass production of two-dimensional materials, mechanical and liquid exfoliation is mainly used [24,25]. Compared to mechanical exfoliation, liquid phase exfoliation (LPE) has several outstanding advantages, such as simple and scalable production, separation of nanosheets of different sizes and thicknesses, further chemical functionalization with other materials, ease of transfer to substrates and creation of thin films. [26,27]. Dispersibility of exfoliated nanosheets TMDs exhibits a relatively low variance among different compounds [28]. As a result, a lot of data obtained for representative TMD materials such as MoS_2_ and WS_2_ can be generalized to the entire TMD class. Liquid phase exfoliation of MoS_2_ can be accomplished by sonication or by shear force. However, a recent study has shown that the sonication method is not suitable for scalable industrial production [29]. In contrast, it has recently been demonstrated that shear separation using simple kitchen blenders [30] and high-shear rotor stator mixers [29] has the potential for large-scale production [31]. The main advantage of this approach is much higher volumes and production rates (reaching values of ~1 mg/min for MoS_2_ [26]) compared to sonication. Moreover, the same method applied to WS_2_ has been reported to achieve throughput rates of up to 0.95 g/h [29].

In addition to physical treatment, LPE requires the choice of solvents and surfactants, which largely determine the separation efficiency and quality of the nanosheets [32,33,34]. The most popular approach is the use of highly polar solvents, including N-methylpyrrolidone (NMP), N,N-dimethylformamide (DMF), and several others [35]. In general, amine-based solvents are the most effective for obtaining exfoliated MoS_2_ nanosheets and their further chemical modification, but their high boiling points as well as high toxicity make solvent removal difficult and carry health risks [36]. Successful exfoliation and stable MoS_2_ dispersions in the less toxic dimethylsulfoxide (DMSO) have also been reported with concentrations similar to those of NMP [37,38]. Other environmentally friendly alternatives are aqueous media with various surfactants, such as sodium cholate [39] and sodium deoxycholate [40] or polymers [41]. The covers nanosheets, which prevents their aggregation, stabilizes the resulting solution, and allows high production rates to be achieved. Moreover, such additives can interact with 2D materials and change their electronic structures and optoelectronic properties [42].

It is also worth mentioning that the inherent photoluminescence (PL) quantum yield (QY) of TMDs is extremely low: MoS_2_ is reported to have a maximum QY of 0.6% [43]. That, in turn, significantly hinders the practical utilization of monolayer and few-layer MoS_2_ in photonic, photocatalytic, and photovoltaic applications. Therefore, the chemical functionalization and surface modification of exfoliated MoS_2_ with various metals, salts, and organic and polymer molecules to enhance its catalytic and photoluminescent properties is an urgent task [44,45,46] and has important practical significance. For example, it was previously demonstrated that using chemical treatment on CVD-grown MoS_2_ samples it is possible to achieve QY as high as 30% [47]. In multilayer graphene introduction of surface functionalization in conjunction with varied stacking configurations presents a compelling avenue for inducing desirable electronic properties. This holds promise for diverse applications, including field-emission displays [48]. Hybridization, in particular, plays a crucial role in chemical functionalization. The attachment of functional groups or chemical species to a material’s surface can lead to their interaction with the underlying atoms or molecules through bonding interactions. These bonding interactions often involve the hybridization of atomic orbitals. Comprehension of the hybridization processes involved is essential for predicting and controlling the properties and reactivity of functionalized materials. Tantardini et al. showed that pressure-induced hybridization changes in silicene enable its use as a field-effect transistor-based pressure sensor [49]. However, until now, studies have not been focused on investigating the dependence of the photoluminescent properties of MoS_2_ solutions on the surfactant concentration, which is of practical importance for the creation of TMD-based organic electronics and the production of thin films by depositing exfoliated flakes on a substrate [50,51,52]. The purpose of this work is to determine the effect of sodium cholate as the most common surfactant on the optical (electronic) properties of exfoliated MoS_2_ in water and DMSO.

## 2. Materials and Methods

### 2.1. Materials

Bulk MoS_2_ powder (<2 μm, 99%, Aldrich, Darmstadt, Germany), dimethyl sulfoxide (DMSO, RusChim, Moscow, Russia), sodium cholate (99%, Sigma-Aldrich, Darmstadt, Germany) and bidistilled water were used as received.

### 2.2. MoS_2_ Liquid Exfoliation

**Figure 1:**
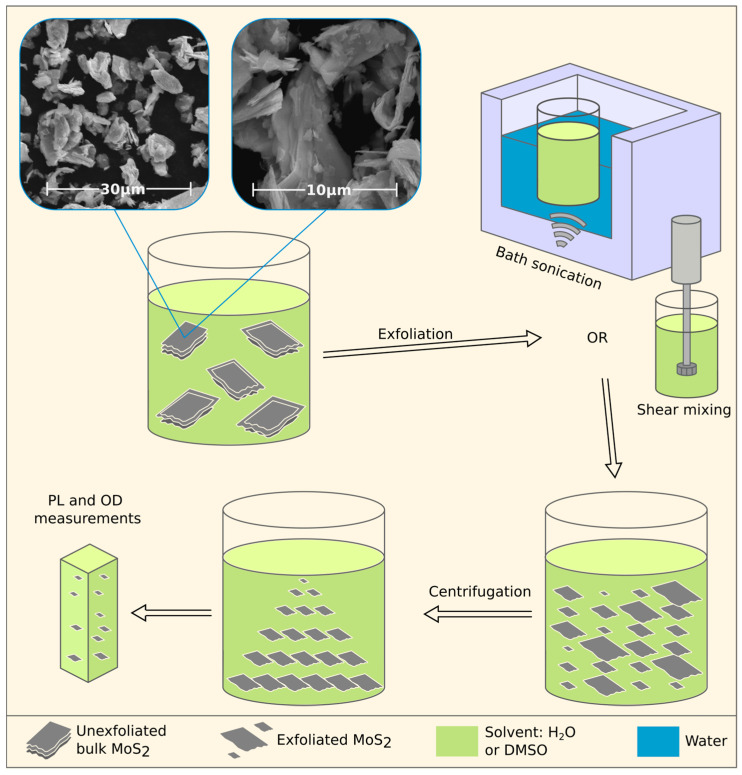
Schematic representation of the synthesis and characterization procedure for MoS_2_ nanosheet dispersions. SEM images represent the pristine powder before the exfoliation.

Direct exfoliation of MoS_2_ into colloidal nanosheets was carried out in an organic solvent (DMSO) and pure water. To improve the dispersing ability of molybdenum disulfide, a surfactant (sodium cholate) was added to the reaction medium, the interaction of which with nanosheets via noncovalent mechanisms plays an important role in the stabilization of dispersions. The dispersions with MoS_2_ concentration of 2.5 mg/mL and with various surfactant (sodium cholate) concentrations in the range from 0.25 to 2.5 mg/mL in pure water and DMSO were prepared using shear force-promoted and sonication-promoted exfoliation. The mixtures were sonicated for 1 h in an ultrasonic (US) bath sonicator (Psb-Gals, Moscow, Russia, 40 kHz, 300 W) or mixed for 2 h with an immersion disperser (Polytron PT 10-35 GT, Kinematica AG, Malters, Switzerland) with a PT-DA 36/2EC-F250 rotating blade at half maximum rotor speed. After exfoliation, the prepared solutions were centrifuged at 1500 rpm for 45 min using a centrifuge (OPN-16, Labtex, Taipei, Taiwan) to remove the unexfoliated MoS_2_ or thick flakes. The upper fraction of each solution was taken for further characterization and analysis. Detailed information about sample preparation is presented in Figure 1 and Table 1.

### 2.3. Characterization

Optical properties of the prepared dispersions were characterized in 10 mm path length cuvettes. Ultraviolet–visible (UV-vis) absorption spectra were recorded with a Cary 5000 UV-Vis-NIR spectrophotometer, and PL spectra were recorded using a Horiba FluroMax 4 spectrofluorometer (HORIBA Jobin Yvon, Kyoto, Japan, Japan–France). Optical density (OD) was detected in the 300–800 nm optical range. Fluorescence spectra were recorded at z-x excitation wavelengths. The emission of fluorescence was detected at 300–360 nm. To diminish agglomeration, all samples were subjected to additional centrifugation before measurements. To minimize possible re-absorption and scattering effect effects [28], all samples from Table 2 were centrifuged using an Eppendorf MiniSpin Centrifuge and diluted to achieve an optical density of less than 0.2 in the wavelength range of 300–360 nm, which corresponds to the excitation wavelengths used for PL measurements. Initial (C_i_) and final (C_f_) concentrations of MoS_2_ and sodium cholate can be found in Table 2.

XPS spectra were acquired on an Axis Ultra DLD spectrometer (Kratos Analytical, Manchester, UK) with a monochromatic Al*K*α radiation source (hν = 1486.69 eV, 150 W). The pass energies of the analyzer were 160 eV for survey spectra and 40 eV for high-resolution scans.

## 3. Results and Discussion

### Optical Properties

**Figure 2:**
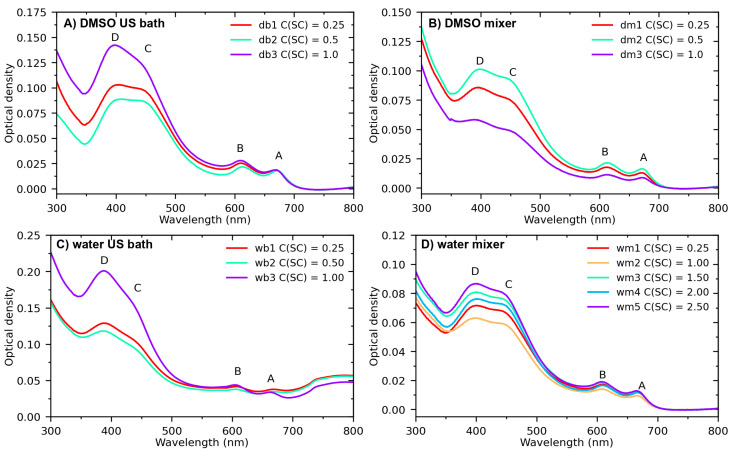
Optical density spectra of dispersions of exfoliated MoS_2_ nanosheets produced via bath sonication in DMSO (**A**) via shear mixing in DMSO (**B**) via bath sonication in water (**C**) via shear mixing in water (**D**) with various initial concentrations of sodium cholate C(SC) as indicated in the legend. Mixing conditions: T = 22 ℃, t = 120 min, C(MoS_2_) = 2.5 mg/mL, and V = 400 mL. Labels in the graphs correspond to two excitonic transitions A (~610 nm), B (~665 nm), and direct transition from deep in the valence band C (~455 nm) and D (~400 nm).

**Figure 3:**
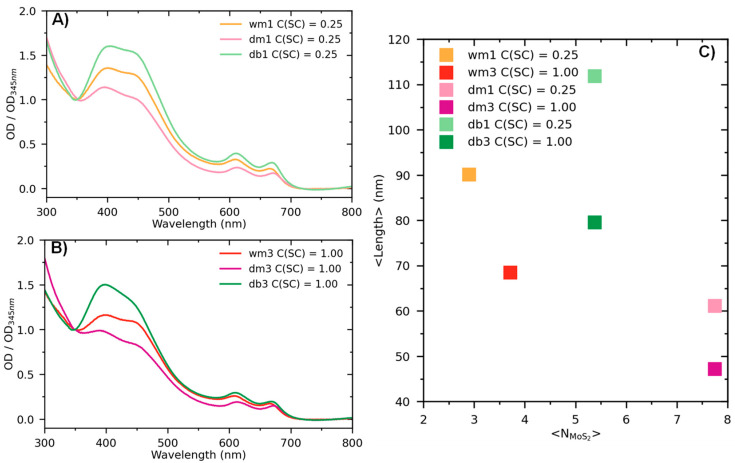
(**A**,**B**) Normalized optical density spectra of dispersions of exfoliated in DMSO/water MoS_2_ nanosheets, prepared via bath sonication/shear mixing with different initial concentrations of sodium cholate C(SC) indicated in the legend. (**C**) MoS_2_ nanosheet dimensions, calculated from the optical density spectra using Equations (1) and (2).

Four series of samples, with different initial concentrations of sodium cholate C(SC), were synthesized using the LPE method: MoS_2_ nanosheets in aqueous and DMSO solutions were produced using ultrasound bath sonication and shear mixing (see details in the Sample Synthesis section). The optical density spectra and photoluminescence spectra are shown in Figure 2 and Figure 3, respectively.

For all four series, the main set of MoS_2_ characteristics was recognized: (i) two excitonic transitions A (~610 nm) and B (~665 nm) characteristic for the 2H polytype of MoS_2_; (ii) broadband topped by peaks C (~455 nm) and D (~400 nm), which are associated with a direct transition from deep in the valence band to the conduction band; and (iii) a pronounced local minimum at ~348 nm, also attributed to transitions from deep in the valence band [53,54,55,56]. Figure 2 clearly shows the influence of the solution type and sodium cholate concentration on the structure of MoS_2_ flakes. Smaller dimensions of MoS_2_ nanosheets were obtained using a mixer (Figure 3). The other two series of samples obtained in DMSO with the mixer (Figure 2B) and in water solution with the US bath (Figure 2C) both at low and high concentrations showed a strong distortion of the optical density spectrum, which indicates an imperfect mechanism of cleavage of individual layers. Broadening of the peaks in Figure 2B points to the absence of few-layer particles and the excess of large micrometer nanoparticles consisting of randomly arranged. The spectrum in Figure 2C demonstrates a strong scattering background, which complicates the further characterization of this series. The thicknesses and lateral sizes of MoS_2_ particles calculated for different dispersions using Equations (1) and (2) (Figure 3C) confirm these suggestions.

It was shown earlier that the parameters of both A- and B-excitons depend on C(SC) [57]. Specifically, the position of A-exciton ($\lambda_{A}$) red-shifts as the number of layers per nanosheet (N) increases, while the intensity of the B-exciton (${OD}_{B}/OD_{348}$) is higher for longer nanosheets [26]. This implies that both the length and the thickness of the nanosheets depend on C(SC) as was shown by J. N. Coleman in [26]. Such behavior has been demonstrated for a number of TMDs and it is attributed to edge confinement effects [19,58,59]. As a result, the optical density spectra provide quantitative information not only about lateral size:

(1) $$L\left(\mu m\right)=\frac{\frac{3.5\;{\mathrm{OD}}_{B}}{{OD}_{348}}-0.14}{11.5-\frac{{\mathrm{OD}}_{B}}{{OD}_{348}}},$$

but also, about the nanosheet thickness:

(2) $$N_{{MoS}_{2}}=2.3\times 10^{36}e^{-54888\lambda_{A}},$$

where ${OD}_{B}$ and ${OD}_{348}$ are the optical densities of peak B and a local minimum at 348 nm. According to C. Backes et al., any arbitrary ratio of peak intensities can be used as a metric for lateral dimensions of nanosheets [57]. For instance, S. Ott et al. have used the ratio between optical density intensities of peaks at 348 and 270 nm [32].

Taking into account the accuracy of the optical density spectra measurements, it was not feasible to find an unambiguous correlation between the preparation method and the average thickness of nanosheets (Figure 3A,B). Mixer-assisted exfoliation of MoS_2_ powder in water solution (wm1) resulted in smaller nanosheet dimensions compared to other synthesis methods with a mean thickness of 3 layers and a mean lateral size of ~80 nm, while bath sonication of the DMSO dispersions led to the formation of rather thick particles (7.5 layers) of shorter lengths of ~55 nm. The dispersions produced in DMSO consistently contained thicker nanosheets than those prepared in water (Figure 3C). However, the reproducibility of the average dimensions was the most successful for MoS_2_ nanosheets in aqueous solutions produced with the shear mixing method. The resulting mean thickness range of 3 to 4 monolayers per nanosheet corresponds to the bandgap values of 1.0–1.2 eV [60]. At the same time, it is crucial to acknowledge that the properties of MoS_2_ become close to bulk as the number of monolayers approaches 10 [61]. Figure 3A,B present the normalized optical density spectra of three series of the dispersions of exfoliated samples with C(SC) concentrations of 0.25 and 1.00 mg/mL. Based on these data, it can be stated that the exfoliation of few-layered MoS_2_ nanosheets in water dispersions is more effective via shear mixing (wm1, orange/red curve in Figure 3B).

**Figure 4:**
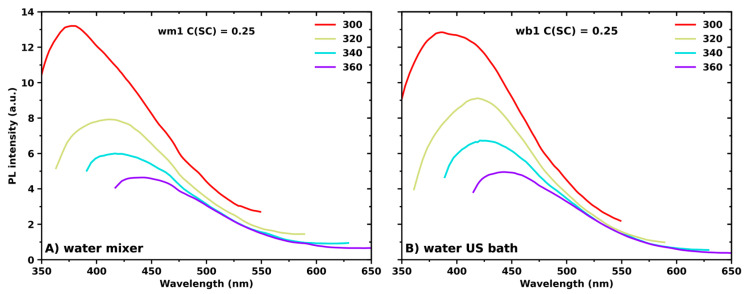
Photoluminescence spectra of MoS_2_ nanosheet dispersions produced via shear mixing (**A**) via bath sonication (**B**) in water with the same initial concentration of SC (C(SC) = 0.25 mg/mL) and MoS_2_ powder (C(MoS_2_) = 2.5 mg/mL).

Figure 4 demonstrates the PL spectra of the samples prepared with the same initial concentration of SC and MoS_2_ powder but with variations in the solvent and LPE method. The analysis of the luminescence properties of MoS_2_ dispersions in DMSO is not possible due to the strong background signal of the solvent [62]. At the same time, for MoS_2_ dispersions in water, the increase in excitation wavelength red-shifted the luminescence spectra (Figure 4A,B). The excitation-dependent luminescence implies the poly-dispersive nature of MoS_2_ aqueous dispersions, which is characteristic of the LPE synthesis method [63]. The observed PL emission is ascribed to the release of energy due to the recombination at the electron (hole) trap [64]. These traps originated from uncompensated positive (negative) charges at the dangling bond of MoS_2_ nanosheets [64]. Dangling bonds are frequently classified as defects, which typically arise from impurities or crystal growth. Taking into consideration the surface-to-volume ratio, it becomes evident that defects exert a more pronounced impact on the properties of monolayers in comparison to bulk materials [65]. For instance, Saigal et al. reported that the nature of at least two discrete emission features in the vicinity of peak A in the PL spectrum of monolayer MoS_2_ on SiO_2_/Si substrates is associated to the recombination of defect-bound excitons [65]. No correlation was found between the synthesis method and luminescence intensity over excitation wavelengths ranging from 300 to 360 nm.

The optical properties were further analyzed for the series of samples prepared using water as a solvent and shear mixing as an LPE synthesis technique.

**Figure 5:**
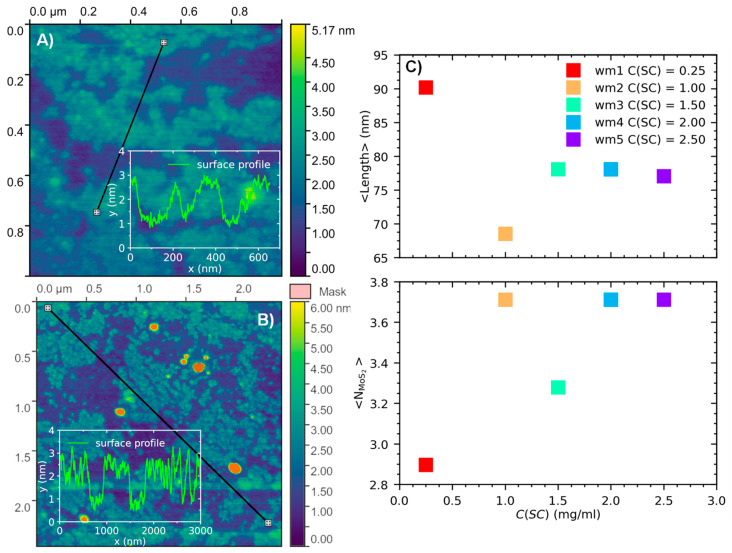
(**A**,**B**) AFM images of typical nanosheets produced using standard mixing parameters with C(SC) = 0 mg/mL and deposited on silicon substrate (**C**) MoS_2_ nanosheet dimensions, calculated from the optical density spectra using Equations (1) and (2).

Dynamic light scattering (DLS) and atomic force microscopy (AFM) measurements (Figure 5A,B) confirmed the MoS_2_ nanosheet dimensions calculated from the optical density spectra using Equations (1) and (2). The nanosheet volume was calculated (V_d_) from the diffusion coefficient using the following equation [66] and found to be 40.5 × 10^3^ nm^3^:

(3) $$D=\frac{13.3\times 10^{-9}}{\eta_{s}^{1.14}V_{d}^{0.589}}$$

where D–diffusion coefficient (cm^2^/s), ν–viscosity of the solvent (mPa/s) and V_d_–LeBas molar volume (cm^3^/mol). Assuming that the largest and the smallest nanosheet mean sizes obtained from the optical density spectra are about 125 nm and 3.5 nm, respectively, the third size derived from Equation (3) the DLS measurements is about 93 nm and, thus, it is indeed intermediate.

For AFM measurements, a surfactant-free dispersion of MoS_2_ nanosheets in water was prepared using standard mixing parameters and then deposited on a silicon wafer. A representative AFM image of the deposited film is shown in Figure 5B. According to the surface profile measurements (see inset Figure 5B), the average thickness of the film did not exceed 5 nm, which is in agreement with the dimension calculated from the optical density (spectra Figure 2D). The films prepared from the SC-contained dispersions demonstrated large organic agglomerates on the substrate surface, which complicates the analysis of the size of MoS_2_ nanosheets.

**Figure 6:**
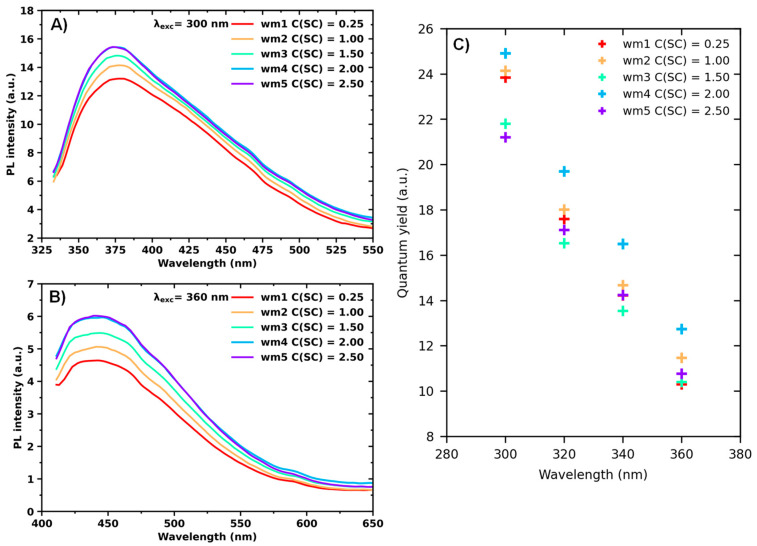
*(***A**,**B**) Photoluminescence spectra of MoS_2_ nanosheet dispersions in aqueous solutions with various initial concentrations of sodium cholate C(SC) indicated in legend prepared by shear mixing. (**C**) Quantum yield of MoS_2_ nanosheet dispersions in aqueous solutions with various initial concentrations of sodium cholate C(SC) indicated in legend prepared by shear mixing.

The PL emission spectra of the dispersions of exfoliated MoS_2_ nanosheets (prepared with various initial concentrations of SC) under different excitation wavelengths (300–360 nm) are shown in Figure 6. The photoluminescence intensity tends to increase for solutions with higher concentrations of SC. This effect can be attributed to the fact that the final concentration of MoS_2_ nanosheets should be higher for dispersions with higher SC concentrations since the addition of surfactant prevents particle reaggregation.

PL and optical density spectra allowed calculation of the QY of the dispersions as integrated fluorescence intensity (the area of the fluorescence spectrum) divided by the optical density at the excitation wavelength. The calculated QY shows no direct correlation with the surfactant concentration. This behavior can be explained by the fact that, with the increasing SC concentration, the increase in optical density has a more pronounced impact on the quantum yield than the increase in integrated PL.

Absolute fluorescent quantum yield was calculated using two different approaches. The first approach uses a spontaneous Raman scattering line of H_2_O (solvent) as an internal standard. Absolute fluorescent QY (Q_s_) can be determined using

(4) $$Q_{u}=Q_{r}\frac{I_{u}}{I_{r}}\frac{{OD}_{r}}{{OD}_{u}}$$

where I_u_ and I_r_ represent integrated fluorescence intensities for the unknown sample and the reference sample, respectively. OD_u_ and OD_r_ denote the optical density values of the unknown and the reference, respectively. The fluorescence of coumarin, which was measured at the same parameters, was used as a reference for the calculation.

The second approach is based on the work and can be calculated as [67]

(5) $$Q_{u}=\frac{n_{s}}{n_{u}}\frac{4\pi \sigma_{RS}}{\sigma_{a}}\frac{I_{u}}{I_{RS}}$$

**Figure 7:**
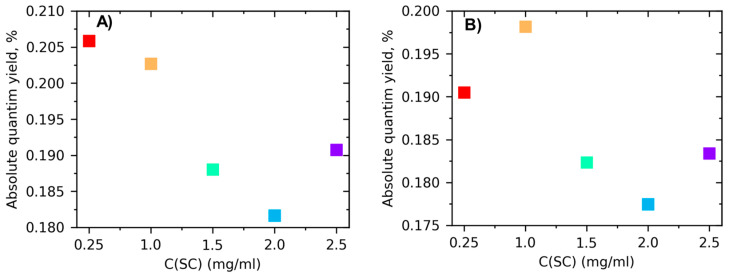
Absolute fluorescent quantum yield of MoS_2_ nanosheet dispersions in aqueous solutions with various initial concentrations of sodium cholate C(SC) indicated in legend prepared by shear mixing. *(***A**) Calculated using Equation (4); (**B**) calculated using Equation (5).

σ_RS_-total differectial Raman scattering cross section of the 3440 cm^−1^ line of water used in calculations σ_RS_(337 nm) = 4.5 × 10^−29^ cm^2^ sr^−1^. Line with the Stokes shift equal to 3444 cm^−1^ and excited at 337 nm will be located at 381 nm, where it was in fact observed during the experiment. n_s_-concentration number of solvent. In our case, it was distilled water n_s_(H_2_O) = 9.4 × 10^18^ cm^−3^. σ_a_-absorption cross section (cm^2^). I_u_ and I_RS_ represent integrated fluorescence intensities for the unknown sample and the 3440 cm^−1^ line of water. The absolute values of fluorescent quantum yield of MoS_2_ nanosheet dispersions in aqueous solutions with various initial concentrations of sodium cholate. The calculated data is presented in Figure 7.

**Figure 8:**
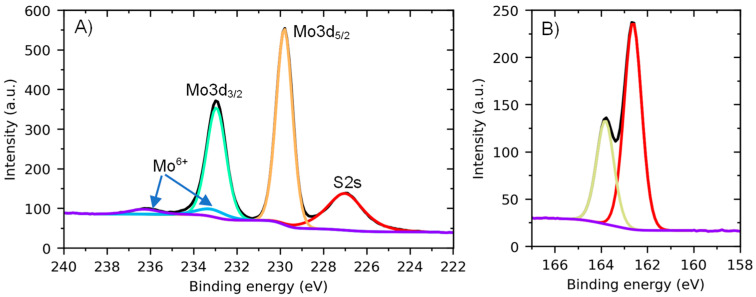
Mo3d–S2s (**A**) and S2p (**B**) XPS spectra of the MoS_2_ nanosheets produced using standard mixing parameters with C(SC) = 0 mg/mL and deposited on a silicon substrate.

The high-resolution Mo3d–S2s XPS spectrum of a typical MoS_2_ nanosheet sample (Figure 8A) shows a strong doublet of Mo3d peaks at a binding energy of 229.8 eV attributed to MoS_2_ and a low-intense doublet at a higher binding energy of about 233 eV that can be assigned to oxidized Mo^6+^ species. At the same time, the S2p spectrum (Figure 8B) shows only a strong doublet at a binding energy of 162.6 eV typical for metal sulfides.

**Figure 9:**
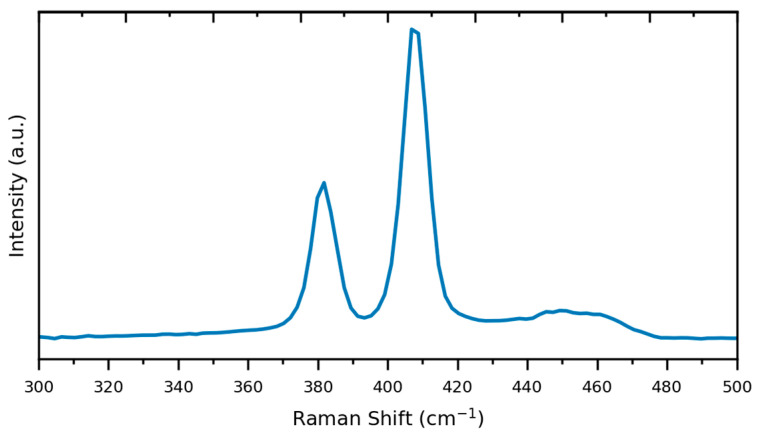
Raman characterization using a 488 nm laser line of typical nanosheets produced using standard mixing parameters and deposited won a silicon substrate.

Raman spectroscopy serves as a valuable tool for the characterization of two-dimensional materials, and this applies to MoS_2_ as well. According to the findings presented in [61], nanosheets containing up to four monolayers can be unequivocally identified. Figure 9 displays the Raman spectra of nanosheets obtained through shear mixing and excited with a 488 nm laser line on a silicon substrate. Unfortunately, the limited resolution of the peaks hindered the determination of the average thickness of the nanosheets using the metrics proposed by [61]. Nevertheless, Figure 9 clearly exhibits two distinct peaks associated with molybdenum disulfide, corresponding to the first-order Raman active modes with E’ and A’ symmetries.

## 4. Conclusions

In this article, we study the optical properties of MoS_2_ modified with one of the most commonly used surfactants, sodium cholate. Our findings illustrate that the shear-exfoliated materials exhibit superior structural and quantum yield properties compared to those produced through sonication. The surfactant concentration was chosen from 0.5 to 2.5 g L^−1^. The average thickness of the produced nanosheets is 3.5 nm, which was confirmed by optical, DLS, and AFM measurements. The concentration of the surfactant does not influence the quantum yield. Sodium cholate does not form chemical bonds with MoS_2_ and does not change the band gap. In conclusion, chemical functionalization in conjunction with shear mixer-assisted LPE has a strong potential as a cheap and easy method for large-scale preparation of exfoliated luminescent MoS_2_ nanosheets. Sodium cholate can be utilized along with other modifiers to stabilize the suspension without altering its optical properties.

## Figures and Tables

**Table 1:** Exfoliated sample preparation.

Solvent Type	Exfoliation Approach	Sample Name	C(SC), mg/mL	C (MoS_2_), mg/mL	Production Time, h	Centrifugation #1
Water	Shear force promoted	wm1	0.25	2.5	2	1500 rpm × 45 min
		wm2	1.0			
		wm3	1.5			
		wm4	2.0			
		wm5	2.5			
	Sonication promoted	wb1	0.25		1	
		wb2	0.5			
		wb3	1			
DMSO	Shear force promoted	dm1	0.25		2	
		dm2	0.5			
		dm3	1			
	Sonication promoted	db1	0.25		1	
		db2	0.5			
		db3	1			

**Table 2:** Final MoS_2_ and sodium cholate concentrations.

Sample Name	C_i_(SC), mg/mL	C_f_(MoS_2_) mg/mL	Centrifugation #2
wm1	0.25	2.5	10 krpm × 6 min
wm2	1.0		
wm3	1.5		
wm4	2.0		
wm5	2.5		
wb1	0.25	1.25	12 krpm × 30 min
wb2	0.5	2.5	
wb3	0.5	2.5	
dm1	0.17	1.67	10 krpm × 5 min
dm2	0.33		
dm3	0.67		
db1	0.015	0.153	10 krpm × 5 min
db2	0.031		
db3	0.061		

## Data Availability

Not applicable.
